# Curcumin and Resveratrol in the Management of Cognitive Disorders: What Is the Clinical Evidence?

**DOI:** 10.3390/molecules21091243

**Published:** 2016-09-17

**Authors:** Gabriela Mazzanti, Silvia Di Giacomo

**Affiliations:** Department of Physiology and Pharmacology, Sapienza—University of Rome, P.le Aldo Moro 5, 00185 Rome, Italy; silvia.digiacomo@uniroma1.it

**Keywords:** polyphenols, curcumin, turmeric, resveratrol, grape wine, dementia, Alzheimer, cognitive disorders, clinical trials

## Abstract

A growing body of in vitro and in vivo evidences shows a possible role of polyphenols in counteracting neurodegeneration: curcumin and resveratrol are attractive substances in this regard. In fact, epidemiological studies highlight a neuroprotective effect of turmeric (rhizome of *Curcuma longa* L.), the main source of curcumin. Moreover, the consumption of red wine, the main source of resveratrol, has been related to a lower risk of developing dementia. In this review, we analyzed the published clinical trials investigating curcumin and resveratrol in the prevention or treatment of cognitive disorders. The ongoing studies were also described, in order to give an overview of the current search on this topic. The results of published trials (five for curcumin, six for resveratrol) are disappointing and do not allow to draw conclusions about the therapeutic or neuroprotective potential of curcumin and resveratrol. These compounds, being capable of interfering with several processes implicated in the early stages of dementia, could be useful in preventing or in slowing down the pathology. To this aim, an early diagnosis using peripheral biomarkers becomes necessary. Furthermore, the potential preventive activity of curcumin and resveratrol should be evaluated in long-term exposure clinical trials, using preparations with high bioavailability and that are well standardized.

## 1. Introduction

Growing evidence suggests that polyphenols have potential health-promoting properties. In fact, these compounds have been associated to pleiotropic biological effects: they are known to behave as potent antioxidants, as direct radical scavengers in the lipid peroxidation, and to interact with a number of signalling targets involved in biological processes, such as carcinogenesis and inflammation [1]. Due to their multiple biological activities, polyphenols have been described as cardio-protective, anti-cancer, anti-microbial, and hepato-protective agents [2]. Evidence also suggests that polyphenols can counteract neurodegeneration so having a possible role in preventing or treating cognitive disorders and neurodegenerative diseases, particularly dementia.

Dementia is a multifactorial syndrome that affects memory, thinking, language, behavior and ability to perform everyday activities. According to the World Alzheimer Report [3], today, dementia affects over 46 million people worldwide and this number is estimated to increase to 131.5 million by 2050 due to increased expectation of life and an aging population [3]. The most common form of dementia is Alzheimer disease (AD) that possibly contributes to 60%–70% of cases, with a greater proportion in the higher age ranges [4]. AD is a multifactorial disease with genetic (70%) and environmental (30%) causes. The familial early-onset form of AD is caused by mutations in genes APP (amyloid precursor protein), PSEN1 (Presenilin 1) and PSEN2 (Presenilin 2). The APOE gene is responsible for the sporadic form of the disease [5]. The pathology initiates in the hippocampus brain region that is involved in memory and learning, then affects the entire brain. Major pathological features of AD include the accumulation of extracellular amyloid plaques and fibrils, intracellular neurofibrillary tangles, as well as chronic inflammation, an abnormal increase of oxidative stress and disruption of cholinergic transmission, including reduced acetylcholine levels in the basal forebrain. The neurodegenerative process leads to synaptic damage, neuronal loss accompanied by astrogliosis and microglial cell proliferation, ultimately leading to brain dysfunction and marked atrophy in susceptible regions of the brain, such as the hippocampus, amygdale and basal forebrain [6,7,8,9].

Amyloid plaques, also known as “senile plaques”, originate from the amyloid beta (Aβ) peptide, following up its aberrant cleavage by β-secretase, of the transmembrane protein amyloid precursor protein (APP), whose function is unclear but thought to be involved in neuronal development. Aβ monomers aggregate into soluble oligomers and coalesce to form fibrils insoluble deposited outside neurons in dense formations, the amyloid plaques, in less dense aggregates as diffuse plaques, and sometimes in the walls of small blood vessels in the brain. Small Aβ oligomers (40 and 42 amino-acids) are particularly toxic to neurons causing membrane damage, Ca^2+^ leakage, oxidative damage, disruptions to insulin signaling pathways and synaptic function, and mitochondrial dysfunction [8,10]. Abnormal Aβ accumulation may be associated with disruption in cholinergic neurotransmission and initiate inflammatory mechanisms that produce reactive oxygen species (ROS). Abnormal release of neurotransmitters such as glutamate contributes to neuronal death and inflammation [11].

In AD, abnormal aggregation of the tau protein (P-tau), a microtubule-associated protein expressed in neurons, is also observed. P-tau acts to stabilize microtubules in the cell cytoskeleton. Like most microtubule-associated proteins, tau protein is normally regulated by phosphorylation; in AD patients, hyperphosphorylated P-tau accumulates as paired helical filaments that in turn aggregate into masses inside nerve cell bodies known as neurofibrillary tangles (NFTs), the other key pathological hallmark of AD [6,12].

There is a direct evidence for free radical oxidative damage in brain of patients with AD [13]. Oxidative stress is associated with various aspects of AD such as metabolic, mitochondrial, metal, and cell cycle abnormalities [14]. Dysregulation of metal homeostasis can lead to the binding of these metal ions to Aβ and acceleration of Aβ aggregation [15]. Oxidative stress is evidenced by lipid peroxidation end products, formation of toxic peroxides, alcohols, free carbonyl, and oxidative modifications in nuclear and mitochondrial DNA [16].

Neuroinflammation is also involved in the complex cascade leading to AD pathology and symptoms. It has been shown that AD is associated with increased levels of cycloxygenase 1 and 2 and of prostaglandins, release of cytokines and chemokines, acute phase reaction, astrocytosis and microgliosis [17]. These pro-inflammatory factors may induce degeneration of normal neurons through upregulation of nuclear factor-κB, mitogen-activated protein kinase, and c-Jun N-terminal kinase [18].

Finally, in patients with AD epigenetic alterations such as changes in DNA methylation, histone modifications, or changes in miRNA expression have been reported. Histone acetyltransferases (HATs) and histone deacetylases (HDACs) promote histone post-translational modifications, which lead to an epigenetic alteration in gene expression. Aberrant regulation of HATs and HDACs in neuronal cells results in pathological consequences such as neurodegeneration [19,20,21].

**Figure 1:**
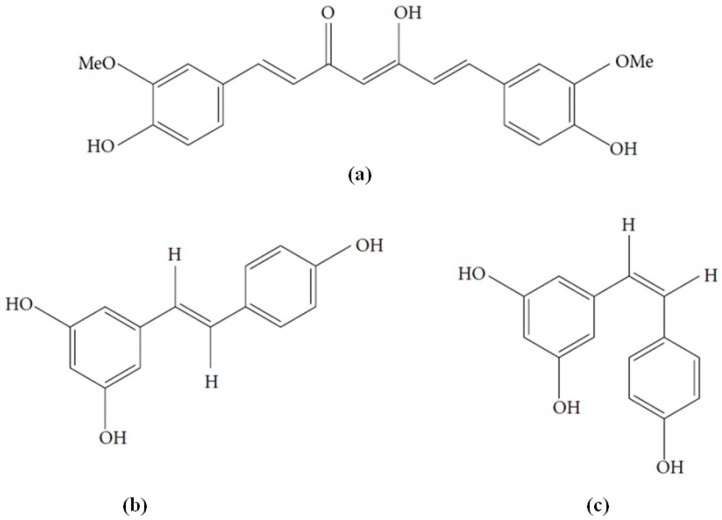
Chemical structures of (**a**) curcumin; (**b**) *trans*-resveratrol and (**c**) *cis*-resveratrol.

In summary, AD appears to be a complex and multifactorial disorder in which extracellular Aβ and intraneuronal hyperphosphorylated tau protein are the hallmark neuropathological features, along with oxidative stress and inflammation. Actually, no current effective disease-modifying treatments are available. Moreover, as Aβ-induced changes are believed to occur a long time before the impairment of cognitive function appears, so strategies to stop or to slow the progression of the disease are of greater importance as is an early diagnosis. Owing to the particular multifactorial nature of the disease, a novel approach consists in evaluating substances having multi-target mechanisms, such as polyphenols. Curcumin and resveratrol are naturally occurring polyphenols of emerging interest in this field (Figure 1). They show close similarity with the presence of several phenolic groups as well as unsaturated carbon chains. Moreover, they share similar biosynthetic pathways in spite of having different biological origins, being 4-hydroxycinnamic acid of the shikimate pathway their starting compound [22]. Curcumin and resveratrol share also other biological properties such as anticancer properties, in which they exert synergistic effects [23,24].

Despite the huge number of pharmacological studies on the potential beneficial effects of curcumin and resveratrol on cognitive disorders, very few of the studies have investigated their efficacy in humans. The present review is aimed not so much at establishing the effectiveness of curcumin and resveratrol in treating cognitive disorder, but rather to give an overview of the studies conducted or in progress using these substances and, possibly, to offer food for thought useful to better directing future research.

## 2. Mechanism of Neuroprotective Action of Curcumin and Resveratrol

Curcumin (1,7-bis(4-hydroxy-3-methoxyphenyl)-1,6-heptadiene-2,5-dione) is a polyphenolic compound obtained by turmeric, the dried rhizome of *Curcuma longa* L. (Fam. Zingiberaceae). Turmeric is the spice that gives curry its yellow color. It has been used in India for thousands of years as a food flavoring and preservative, and as a herbal remedy [8,25]. It is known in traditional medicine for its antiinflammatory, antioxidant, anticarcinogenic, hepatoprotective, cardioprotective, vasodilator, hypoglycemic, and anti-arthritic properties [26]. Turmeric has also been reputed to possess neuroprotective effects [27,28]; in fact, a lower prevalence of AD has been observed in Indian people, who regularly consume turmeric as a part of curry [29,30,31]. Moreover, some epidemiological studies support a link between dietary curry consumption and improved cognitive performance in elderly populations [32]. It is believed that the properties of turmeric are mainly due to its curcumin content [26].

Resveratrol (3,5,4′-trihydroxystilbene) belongs to a family of polyphenolic compounds known as stilbenes, a group of widespread plant secondary metabolites. Resveratrol is also one of the phytoalexins, a group of low-molecular-mass substances with antimicrobial activity, produced by plants as a defence response to some exogenous stimuli, such as UV radiation, chemical stressors, and particularly, microbial infections [33]. Sources of resveratrol in human diet are mainly peanuts, pistachios, berries and grapes; however, the most important dietary source of resveratrol is red wine [34]. The compound exists in two isomeric forms, the *trans*-isomer occurs in the berry skins of most grape cultivars, and its synthesis is stimulated by UV light, injury, and fungal infection. *Cis*-isomer is produced by UV irradiation of the *trans*-isomer; it is generally absent or only slightly detectable in grapes but originates from its *trans*-isomer during vinification, so both forms are present in variable amounts in commercial wines [33,35]. Most research on resveratrol concerns the *trans*-isomer owing to its natural presence in grapes and its greater stability [36].

The research interest in the therapeutic relevance of resveratrol has originated from its association with the “French Paradox” in the early 1990s [37]. It has been reported that the consumption of red wine on a regular basis may be related to a lower risk of developing dementia, such as AD and vascular dementia [38]. Orgogozo et al. [39] have shown a positive correlation between a moderate consumption of red wine and a decreased incidence of dementia. This protective effect is most likely due to the presence in wine of phenolic compounds, in particular resveratrol [1,33].

The neuroprotective potential of curcumin and resveratrol has been highlighted by in vitro and in vivo studies in which the compounds seem to slow down the progression of AD by multiple mechanisms. Both compounds possess anti-amyloidogenic effects. Curcumin has been shown to inhibit the formation and extension of neurotoxic Aβ fibrils, and to destabilize preformed Aβ fibrils [40,41]. A recent study conducted by Fu and coworkers [42] found that curcumin interacts with the *N*-terminus of Aβ_1-42_ monomers and prevents the enlargement of oligomers from 1–2 nm to 3–5 nm. In another study, it was found that curcumin induces significant conformational changes in the Asp-23-Lys-28 salt bridge region and near the Aβ_1-42_ C terminus. Mithu et al. [43] also showed, by using electron microscopy, that curcumin was able to disrupt the Aβ_1-42_ fibrils architecture. Furthermore, the preventive administration of curcumin in Sprague-Dawley rats infused with Aβ_40_ and Aβ_42_ to induce neurodegeneration and Aβ deposits improved memory function [44]. These results were confirmed by a later study carried out by Ahmed et al. [45] who demonstrated that curcumin increased the expression levels of genes involved in synaptic plasticity, such as synaptophysin. More recently, Belviranli et al. [46] found that supplementation with curcumin for 12 days in aged female rat improves spatial memory. Also, resveratrol is reported to reduce the level of secreted or intracellular Aβ peptides by modulating the proteasome [47]. It may act indirectly by selectively stimulating the proteasomal degradation of critical regulators of Aβ clearance. The protective effect of resveratrol was also associated with the activation of protein kinase C, which stimulates α-secretase enzyme and consequently the non-amyloidogenic pathway, resulting in a reduction in the Aβ production [48]. In addition, a direct action of resveratrol towards Aβ plaques was also observed. In fact, it was shown to directly interact with Aβ peptides by inhibiting and destabilizing the formation of Aβ_1-42_ fibrils [49]. Conversely, Granzotto and Zatta [50] reported that resveratrol was unable to prevent Aβ fibril formation in human neuroblastoma cells exposed to Aβ suggesting that resveratrol acts not through anti-aggregative pathways but mainly via its scavenging properties.

Curcumin and resveratrol have potent antioxidant activity that could have a role in preventing neurodegeneration in AD [51]. Cognitive deficits were shown to be associated with higher levels of reactive oxygen (ROS) and nitrogen species (RNS). Moreover, it seems that oxidative stress precedes the formation of senile plaques [52]. The brain possesses a relative deficiency of antioxidant systems and is very prone to oxidative imbalance and consequently to oxidative damage. The source of oxidant species in the AD brain may include unbound transition metals, damaged mitochondria and Aβ peptides themselves [51]. In vitro studies showed that curcumin scavenges nitric oxide (NO) radicals and protects the brain from lipid peroxidation [53]. It also prevents the DNA-oxidative damage by scavenging the hydroxyl radicals [54]. Curcumin was shown to bind Cu^2+^ and Fe^2+^ ions, which are involved in the exacerbation of Aβ aggregation and in the subsequent oxidative damage in the AD brain [55]. Recently, these results were confirmed by Banerjee [56], which demonstrated that curcumin is able to give complexes with Cu^2+^ and/or Zn^2+^ and consequently to inhibit the formation of β-sheet-rich Aβ protofibrils from less structured oligomers. Curcumin also activates glutathione S-transferase [57], partially restores glutathione content in the brain [58], and induces the antioxidant enzyme heme oxygenase-1 (HO-1), which has been shown to increase tolerance of the brain to stresses [59]. González-Reyes et al. [60] showed that the pre-treatment of cerebellar granule neurons of rats with curcumin effectively increased the HO-1 expression and GSH levels, by inducing nuclear factor (erythroid-derived 2)-like 2 (Nrf2) translocation into the nucleus. Besides, in vivo studies showed the ability of curcumin to reduce the brain levels of oxidized proteins containing carbonyl groups [41]. Begum et al. [61] observed a lower protein oxidation in the curcumin-treated Tg2576 mice and suggested that the dienone bridge, present in the chemical structure of curcumin, is necessary for this. In an in vivo study conducted by Belviranli et al. [46], a decrease of MDA levels in brain tissue was observed after curcumin supplementation. Also, resveratrol may block oxidative stress involved in the pathogenesis of AD. It scavenges free radicals, protects neurons and microglia [62,63] and attenuates Aβ-induced intracellular ROS accumulation [64]. Kwon et al. [65] found that the treatment of a murine HT22 hippocampal cell line with resveratrol attenuated ROS production and mitochondrial membrane-potential disruption; moreover, it restored the normal levels of GSH depleted by the Aβ_1-42_. It is known that beta amyloid induces production of radical oxygen species and oxidative stress in neuronal cells, which in turn upregulates BACE-1 expression and beta amyloid levels, thereby propagating oxidative stress and increasing neuronal injury. Resveratrol is able to attenuate Aβ-induced intracellular ROS accumulation [64,66]. It also induces the up-regulation of cellular antioxidants (i.e., glutathione) and the gene expression of phase 2 enzymes, protects against oxidative and electrophilic injury [67], and, like curcumin, it potentiates the HO-1 pathway [68]. Chronic administration of this compound also significantly reduces the elevated levels of malondialdehyde in rats [69,70].

Another important pathological hallmark of AD is represented by brain inflammation. Inflammatory mediators such as cytokines and chemokines released by activated cells (microglia, astrocytes, macrophages and lymphocytes) contribute to the neuronal damage and enhance Aβ formation [71]. Curcumin has been reported to regulate inflammatory responses by suppressing the activity of the transcription factors, nuclear factor kappa-light-chain-enhancer of activated B cells (NF-κB) and activator protein-1 [72,73,74]. Additionally, curcumin blocks the induction of inducible nitric oxide synthase (iNOS) and inhibits lipoxygenase and cyclooxygenase-2 (COX-2) [72,73,74]. In vitro and in vivo studies also showed that it inhibits TNF-α, IL-1, -2, -6, -8, and -12 [75,76]. Resveratrol as well was shown to interfere with the neuroinflammatory process [77]. Particularly, it suppresses the activation of astrocytes and microglia [63,78,79], TNF-α and NO production by inhibiting NF-κB activation and p38 mitogen-activated protein kinase (MAPK) phosphorylation [79,80]. Resveratrol also blocks the expression of COX-2 and iNOS [81]. In a recent study carried out by Huang and coworkers [82], resveratrol treatment was shown to reverse the Aβ-induced iNOS overexpression. This compound is also able to reduce the expression of prostaglandin E synthase-1 [63]. Furthermore, the anti-inflammatory effects of resveratrol are due to the inhibition of TNF-α, IL-1β, and IL-6 expression [80,83], and STAT1 and STAT3 phosphorylation [84].

At last, the interference with epigenetic mechanisms has also been ascribed to curcumin and resveratrol. Epigenetic mechanisms modulate gene expression patterns without affecting the DNA sequence. Gene expression can be activated or silenced via epigenetic regulations; so, epigenetic changes may mediate the differences in risk for certain diseases [85]. Recently, it has been shown that curcumin is a potent inhibitor of histone acetyltransferases (HAT) [86] and DNA methyltransferase (DNMT1) [87]. These enzymes control the expression of genes involved in AD pathogenesis [88]. DNA methylation and histone post-translational modifications are crucial for synaptic plasticity, learning, memory, neuronal survival [89] and repair [90]. Also, resveratrol is able to inhibit DNMT activity [87] and to induce histone post-translational modifications. Indeed, it contributes to improvement of cognitive functions by activating SIRT1, a member of nicotinamide adenine dinucleotide (NAD+)-dependent deacetylases family [91,92].

## 3. Methods

A systematic research of the literature was carried out on PubMed, MedlinePlus and Google Scholar databases using the key words: curcumin, curcuminoids, turmeric, *Curcuma longa*, resveratrol, grapes, stilbenes, and wine. Each term was matched with the key words: neurodegenerative disorders, cognitive impairment, cognitive disorders, cognitive function, memory, learning, brain disease, dementia, Alzheimer, neuroprotection, and clinical trials. Furthermore, in order to find ongoing studies on curcumin and resveratrol in cognitive disorders, some accessible databases on clinical trials [93,94] were examined, using the methodology described above. No time or language restrictions were applied to the search strategy.

As yet stated, the aim of present review was to give an overview of the studies conducted or in progress using these substances, so all studies identified (both published and ongoing) in which curcumin and resveratrol are used in prevention, in treatment and in diagnosis of cognitive disorders, were selected and included in the review. Some studies carried out to evaluate the capability of the substances to increase the cerebral blood flow were included too, because this parameter is associated with improved cognitive performance [95]. Published studies were retrieved and carefully analyzed, also to acquire further relevant references. Ongoing studies were carefully examined and described, too.

## 4. Results

Our search identified five published studies for curcumin and six for resveratrol, along with 10 ongoing clinical trials on curcumin and nine on resveratrol. Following, the results of published studies will be described along with the characteristics of the ongoing ones (Table 1 and Table 2).

### 4.1. Curcumin

Baum et al. [96] carried out a six-month randomized, double blind, placebo-controlled clinical trial on 34 Chinese patients of both sexes, ≥50 years old, with progressive decline in memory and cognitive function for 6 months, and diagnosis of probable or possible AD, to examine the curcumin safety and effects on biochemical parameters and cognitive function. Subjects received curcumin 1 g/day or 4 g/day; they were also given an additional treatment consisting in 120 mg/day of standardized gingko leaf extract. Patients treated with anticoagulant or antiplatelet agents or with bleeding risk factors were excluded. The main outcome measures were the Mini-Mental Status Examination (MMSE) score at baseline and at 6 months, plasma isoprostanes iPF2α-III and serum Aβ (at 0, 1, and 6 months); plasma levels of curcumin and its metabolites were also measured. No significant differences in MMSE score between treatments (1 g/day and 4 g/day) and placebo were observed, and neither was a reduction in serum Aβ_40_ levels nor differences in plasma isoprostanes iPF2α-III found. Plasma levels of curcuminoids, measured at 1 month, did not differ significantly between 1 g/day and 4 g/day groups so they were pooled and the results were as follows (in nanomolar): 250 ± 80 curcumin, 150 ± 50 demetoxycurcumin, 90 ± 30 bisdemetoxycurcumin, 440 ± 100 tetrahydrocurcumin. No differences in adverse events between curcumin groups and placebo were reported.

Ringman et al. [97] performed a randomized, double blind, placebo-controlled clinical trial on 36 subjects with mild-to-moderate AD. Patients were treated with Curcumin C3 Complex^®^ (95% curcuminoids with curcumin 70%–80%, demethoxycurcumin 15%–25%, bisdemethoxycurcumin 2.5%–6.5%) at 2 g/day or 4 g/day for 24 weeks with an open-label extension to 48 weeks. Other medications such as acetylcholinesterase inhibitors and memantine were allowed, instead antioxidant, anticoagulant or antiplatelet drugs, including *Ginkgo biloba*, were not allowed. The purpose of the study was to acquire data on safety and tolerability and preliminary data on efficacy with regard to cognition, by measuring the incidence of adverse events, changes in clinical laboratory tests and Alzheimer’s Disease Assessment Scale-Cognitive (ADAS-Cog) subscale, Neuropsychiatric Inventory (NPI), Alzheimer’s Disease Co-operative Study-Activities of Daily Living (ADCS-ADL), MMSE score, plasma levels of Aβ_1-40_ and Aβ_1-42_, cerebrospinal fluid levels of Aβ_1-42_, t-tau, p-tau181 and F_2_-isoprostanes. Plasma levels of curcumin and its metabolites were also measured. No significant differences between curcumin and placebo in ADAS-Cog, NPI, ADCS-ADL or MMSE score were registered, as well as no differences between treatment groups in biomarker efficacy measures. Plasma levels of native curcumin and tetrahydrocurcumin, measured at a 24-week visit and 3 h after medication, were 7.76 ± 3.23 and 3.73 ± 2.0 ng/mL, respectively. The levels of glucuronidated curcumin and tetrahydrocurcumin were 96.05 ± 26 ng/mL and 298.2 ± 140.04 ng/mL. Levels of native curcumin were undetectable in the cerebrospinal fluid. Curcumin was generally well tolerated. On the whole, authors were unable to demonstrate clinical or biochemical evidence of curcumin efficacy against AD.

Hishikawa et al. [98] reported three cases of patients (79, 83, and 84 years old, respectively) with AD whose behavioral symptoms were improved remarkably as a result of turmeric treatment. Patients received turmeric 764 mg/day (curcumin 100 mg/day) for more than 1 year. After 12 weeks of treatment, all three patients experienced a reduction (≥50%) of the Japanese version of Neuropsychiatric Inventory-brief Questionnaire (NPI-Q) score and the burden of caregivers was reduced (38%–86%), too. Particularly, agitation, apathy, anxiety, and irritability symptoms were relieved. One patient also increased his MMSE score from 12/30 to 17/30, improving calculation, concentration, transcription of the figure, and spontaneous writing. Of note, two patients were on donepezil treatment before starting curcumin.

Cox and colleagues [99] performed a randomized, double-bind, placebo controlled, phase 3/4 trial in healthy older subjects using Longvida^®^ Optimized Curcumin, in dose of 400 mg, containing approximately 80 mg curcumin in a solid lipid formulation. Participants (aged 60–85 years) were randomly assigned to either curcumin (*n* = 30) or placebo (*n* = 30) treatment groups. The acute (1 and 3 h after a single dose), chronic (4 weeks) and acute-on-chronic (1 and 3 h after single dose following chronic treatment) effects of curcumin preparation on cognitive function, mood and blood biomarkers were examined. The authors reported significantly improved performance in sustained attention and working memory tasks, compared with placebo, one hour after administration of curcumin. Following chronic treatment, working memory and mood (general fatigue and change in state calmness, contentedness and fatigue induced by psychological stress) were significantly improved. A significant acute-on-chronic treatment effect on alertness and contentedness was also observed. Curcumin was associated with significant reduction of total and LDL cholesterol levels. Curcumin treatment was well tolerated and did not significantly impact any of the examined hematological safety measures.

A recent randomized, double-bind, placebo controlled trial (ACTRN12611000437965) was carried out on healthy older adults, to evaluate the potential efficacy of BCM-95^®^CG (Biocurcumax™), a standardised extract of *Curcuma longa* L. (88% curcuminoids and 7% volatile oil), in preventing cognitive decline [100]. One hundred and sixty healthy subjects (40–90 years aged) were selected for the study and randomly divided into two groups: an experimental group, taking Biocurcumax™ 1500 mg/day (1 capsule 500 mg three times a day), and a control group, taking a placebo (roasted rice powder). Sixty four subjects (23 in the placebo group and 41 in the curcumin group) did not complete the study for various reasons (ineligibility to remain in the study, suspect adverse reaction, intervention non-compliance, etc.) so 96 participants were included in the final analysis. The study lasted 12 months during which subjects were evaluated at the baseline and at the 6- and 12-months follow-ups. Mood was assessed by administration of the Depression Anxiety Stress Scale (DASS) [127]. Cognitive measures were obtained by a battery of cognitive tests for general cognitive function (MoCA) [128], verbal fluency, percentual motor speed, psychomotor speed, working memory, executive functions and visual memory. Blood pressure and weight were checked at baseline and every 3 months. No differences were observed in all clinical measures as well as for cognitive measures except for a significant interaction between time and treatment group in the MoCA test that, however, was due to a decline in general cognitive function of the placebo group at 6 months that was not observed in curcumin treatment group.

Besides the published ones, a number of studies of curcumin supplementation in healthy older people or in patients with Mild Cognitive Impairment (MCI) or AD are still underway or completed but results are not yet available.

A clinical trial (NCT00595582) [101] aimed at determining the efficacy of curcumin in the treatment of MCI or mild AD has been registered at the U.S. National Institutes of Health. Ten subjects (both sexes, ≥55 years old) already enrolled in another study (NCT00243451) [129] have been included in this clinical trial. They had to receive 5.4 g/day of curcumin in combination with bioperine, to improve curcumin bioavailability, for 24 months. Primary outcome was to determine if curcumin had an effect on neuropsychological scores, while determining if curcumin impacted the metabolic lesions found in patients who had MCI or might develop MCI was the secondary outcome. Unfortunately, none of the patients terminated the study. In particular, two out of 10 interrupted the study because of adverse effects (dyspepsia).

Another randomized, double-blind, placebo-controlled phase 2 study (NCT01001637) [102] has been designated to evaluate the efficacy and safety of the high-bioavailability curcumin formulation (Longvida^®^) in AD. The study planned to enrol 26 patients (both sexes ≥50 years old) with AD, who had to be treated with placebo, 4 g/day or 6 g/day of curcumin supplementation for 2 months. The main outcomes were to determine if curcumin formulation affects cognitive function in Alzheimer’s patients, based on mental exams, and blood concentrations of Aβ. The recruitment status of this study is unknown.

A larger phase 2 clinical trial (NCT01383161) [103] has been designed to determine the effects of the curcumin supplement on age-related cognitive impairment, after 18 months’ treatment. The investigators will study 132 subjects with MCI (aged 50–90 years), which will be randomly assigned to placebo or curcumin group (six 465 mg Theracurmin™ capsules/day, containing 30 mg of curcumin each). The main outcomes will be a change in cognitive testing results, in level of inflammatory markers in blood, and in the amount of brain amyloid protein. This study is ongoing, but is not recruiting participants.

Additionally, a randomized, double-blind, placebo-controlled phase 2 study (NCT01811381) [104] will evaluate the clinical benefits of curcumin alone or in combination with yoga in 80 individuals with MCI, 55–90 years old. For the first 6 months of the study, subjects will take either 800 mg of curcumin (Longvida^®^ formulation) or placebo, before meals. Over the second 6 months of study, the curcumin and placebo groups will be further divided into groups receiving training in either aerobic or non-aerobic yoga (attendance at 2 classes of 1 h duration and 2 home practices of 30 min duration per week) to determine the synergism between curcumin supplementation and aerobic exercise. Primary outcome will be to determine if curcumin (first six month period), alone or associated to aerobic yoga (second six month period), reduces the levels of blood biomarkers for MCI. Secondary outcome will be to evaluate imaging changes in all subjects and adverse events. This study is currently recruiting participants.

Several clinical trials are also underway in Australia. The randomized, double-blind, placebo-controlled, phase 3/4 clinical trial ACTRN12616000484448 [105] will soon start to recruit participants. This study will investigate the effects of 12 weeks of treatment with a bioavailability enhanced curcumin supplement on cognitive function, mood and wellbeing in healthy older adults (*n* = 80; aged 50–85 years). Participants will receive 400 mg of Longvida^®^ Optimized Curcumin (containing about 80–90 mg curcumin) daily. The study will also evaluate the effects of curcumin on cardiovascular function and a range of blood markers of health, to better understand how cognitive and mood benefits might be achieved. In a subset of participants, the effects of curcumin on brain function will be explored by functional Magnetic Resonance Imaging (fMRI). Finally, the study will investigate whether genetic differences can influence the effects of curcumin.

Another randomized, double-blind, placebo-controlled, phase 2 study (ACTRN12613000681752) [106] is in progress in Australia. This clinical trial will investigate the role of curcumin in preventing AD. Participants (*n* = 100; 65–90 years old) assessed as at high risk of AD will be assigned to placebo or curcumin (Biocurcumax™) group. The latter will receive 500 mg daily of curcumin for 2 weeks, progressing to 500 mg twice daily (1000 mg/daily) for another 2 weeks, then 500 mg three times daily, (1500 mg) until the end of the study (12 months). Primarily, the ability of Biocurcumax to positively alter AD-related blood biomarker profiles compared with placebo will be investigated, secondarily the possible increase in brain glucose utilisation, as measured by FDG-PET, and the correlation with the improvement in cognitive functioning will be studied. Currently, the recruitment of participants is in progress. Present clinical trial includes also a sub study (ACTRN12614001024639) [107], which will be performed in 48 subjects (24 healthy older people and 24 with MCI) to examine the influence of curcumin on expression of inflammatory genetic markers, by measuring associated proteins in the blood, and on existing lifestyle patterns including sleep, activity levels and nutrition.

In addition, three clinical trials have been designed to assess the possible use of curcumin in the early diagnosis of AD. It has been proposed, in fact, that beta-amyloid plaques may first appear in the retina, at the back of the eye, before they are detectable in the brain. Curcumin has molecular and optical properties that allow to image amyloid plaques using a specialized eye camera, and hence to detect Alzheimer’s disease earlier. In these open, not randomized phase 2 or phase 2/3 studies, all groups receive the same intervention, i.e., 20 g of Longvida^®^ (equivalent to 4 g of curcumin) along with Vitamin E supplement capsules, equivalent to 500 IU daily, for seven consecutive days; participants will be asked to have eye imaging done before and after taking curcumin for 7 days. The trial ACTRN12613000367741 [108] includes three groups of subjects: with AD, with MCI and healthy controls. The association between retinal imaging and brain amyloid imaging will be determined in AD and in MCI in comparison with the healthy controls. The second diagnosis trial (ACTRN12615000465550) [109] is recruiting subjects with MCI and healthy controls. Participants must have completed curcumin based fluorescence retinal imaging under the ACTRN12613000367741 study (parent study) [108] within the previous 21 months. The primary endpoint will be to evaluate the ability to detect changes over time in retinal Aβ plaque burden, and at this aim the results from participants with MCI will be compared with the results from the healthy controls and, in addition, with the participants’ results from the parent study. Finally, ACTRN12615000677505 [110] has been designed to investigate the presence/absence of retinal amyloid plaques in a middle-aged control cohort (40–60 years). Furthermore, the comparison between retinal Aβ protein plaque burden with brain Aβ protein plaque burden will be done. Currently, the present study is not recruiting subjects.

### 4.2. Resveratrol

Six studies, aimed at evaluating the effects of resveratrol on cognitive function in humans, were retrieved (see Table 2).

In 2010, Kennedy et al. [111] performed a randomized double-blind, placebo-controlled, crossover study to investigate the possibility that single oral doses of resveratrol modulate mental function and increase cerebral blood flow (CBF) in the frontal cortex of healthy humans. Cognitive performance was measured by a battery of tasks and the cerebral blood flow in the prefrontal cortex was detected by Near-Infrared Spectroscopy (NIRS), during the tasks. The 24 subjects enrolled (age 18–25 years) received three single dose treatments: placebo, 250 mg *trans*-resveratrol, and 500 mg *trans*-resveratrol. The bioavailability of resveratrol and its metabolites (resveratrol glucuronide and resveratrol sulfate) at time points relevant to the CBF/cognitive task assessment, were also evaluated in a separate cohort of 9 healthy young adults. The 500 mg resveratrol supplementation significantly increased CBF and hemoglobin status in the period of 45–81 min following administration. Also, the 250 mg resveratrol dose increased CBF compared to placebo, although in a lesser extent and at fewer time points than the higher dose, suggesting that resveratrol improves CBF in a dose-dependent manner. The peak plasma levels after treatment with 250 and 500 mg of trans-resveratrol were 5.65 ng/mL and 14.4 ng/mL respectively; at the same doses, peak plasma levels of *trans*-resveratrol glucuronide were about 30 ng/mL and 200 ng/mL, respectively, while those of *trans*-resveratrol sulfate were about 300 ng/mL and 750 ng/mL, respectively. Despite increased blood flow in both treatment groups, resveratrol did not enhance cognitive function.

A further randomized, double-blind, placebo controlled, crossover clinical trial has been designed to evaluate the effects of resveratrol on circulatory function and cognitive performance in obese adults [112]. Twenty-eight subjects of both sexes (40–75 years old), obese but otherwise healthy, were enrolled. Participants were randomized to consume a capsule containing either 75 mg of resveratrol (resVida) or a color-matched placebo daily for 6 weeks. Then, participants were crossed over to an alternate dose for another 6 weeks. The assessments were done at baseline, at week 6, and at week 12. Moreover, following the assessments in week 6 and 12, participants consumed a single additional dose of the supplement and further assessments were performed 1 h after consumption. The primary outcome was to measure the degree of change in vasodilator function assessed by flow mediated dilatation (FMD) in the brachial artery. Secondary outcomes were the ability to maintain attention and concentration during the test (measured by the Stroop test), supine blood pressure, heart rate and arterial compliance. Resveratrol supplementation was found to be well tolerated and induced a 23% increase in FMD compared with placebo. Moreover, a single dose of resveratrol (75 mg) following chronic resveratrol supplementation resulted in an acute FMD response 35% greater than placebo. However, blood pressure, arterial compliance, and all components of the Stroop Color-Word Test were unaffected by chronic resveratrol supplementation.

Witte and colleagues in 2014 [113] carried out a double-blind placebo-controlled study aimed to assess the ability of resveratrol, given over 26 weeks, to enhance the cognitive performance. Forty-six overweight older adults (50–80 years old) were recruited and randomly divided in: treatment group, receiving 200 mg/day of resveratrol in a formulation with quercetin (320 mg) to increase its bioavailability and control group, receiving placebo. Memory retention was evaluated by a battery of tasks; volume, microstructure, and functional connectivity of the hippocampus were explored by magnetic resonance imaging (MRI). Further anthropometric measures were: glucose and lipid metabolism, inflammation, neurotrophic factors, and vascular parameters. Resveratrol supplementation induced retention of memory and improved the functional connectivity between hippocampus and frontal, parietal, and occipital areas, in healthy older overweight adults compared with placebo. The changes in resting-state functional connectivity networks of the hippocampus after resveratrol intake were linked with behavioral improvements. Also, glucose metabolism was improved and this may account for some of the beneficial effects of resveratrol on neuronal function. Finally, a significant reduction of body fat and increases in leptin compared with placebo was observed.

In 2014, Wightman et al. [114] performed a randomized, double-blind, placebo controlled, cross-over study investigating the effects of 250 mg resveratrol administered alone or co-supplemented with 20 mg piperine. The aim was to ascertain whether piperine is able to enhance the bioefficacy of resveratrol with regard to CBF and cognitive performance in a cohort of 23 healthy adults (age 19–34 years). Plasma concentrations of resveratrol were also measured in a separate cohort of 6 male adults, to investigate whether bioavailability correlated with bioefficacy. Participants were given placebo, *trans*-resveratrol (250 mg) and *trans*-resveratrol with 20 mg piperine on separate days, at least a week apart. Whereas 250 mg of orally administered *trans*-resveratrol had no significant effects on overall CBF during the performance of cognitively demanding tasks, co-administration of the same dose of resveratrol with 20 mg piperine resulted in significantly increased CBF for the duration of the 40 min post-dose task period. Cognitive function, mood and blood pressure were not affected. In subjects treated with resveratrol alone plasma concentrations of total resveratrol metabolites ranged from 2 to 18.2 μM. Resveratrol 3-*O*-sulphate was the major metabolite, contributing 59%–81% of total metabolites. The 4′-*O*-glucuronide and the 3-*O*-glucuronide forms made roughly equal contribution to the remaining metabolites. No significant differences were observed in the plasma concentrations of resveratrol in subjects receiving resveratrol plus piperine, so suggesting that piperine increases the efficacy of resveratrol on CBF by potentiating its vasorelaxant properties. Despite the piperine-mediated potentiation of CBF during task performance, no effects were found on cognitive performance.

Another randomized, placebo-controlled, double-blind, multicenter 52-week phase 2 trial of resveratrol in individuals with mild-to-moderate AD was conducted between June 2012 and March 2014 to assess the safety and efficacy of resveratrol [115]. Participants (*n* = 119, >49 years old) were recruited and randomly divided in placebo group and resveratrol group (500 mg orally once daily with dose escalation by 500 mg increments every 13 weeks, ending with 1000 mg twice daily). Pharmacokinetic studies were performed on a subset (*n* = 15) at baseline and weeks 13, 26, 39, and 52. The trial showed that resveratrol, also at the higher oral dose, was safe and well-tolerated. In fact, the most common AEs were nausea and diarrhea, but their frequency was similar to placebo. In the treatment group, weight loss was also highlighted. The levels of Aβ_40_ in the cerebrospinal fluid (CSF) and plasma declined more in the placebo group than the resveratrol-treated group with a significant difference at week 52 (note that Aβ_40_ levels decline as dementia advances). No effects of drug treatment were found on plasma Aβ_42_, CSF Aβ_42_, CSF tau, CSF phospho-tau 181, hippocampal volume, entorhinal cortex thickness, MMSE score, Clinical Dementia Rating (CDR) score, ADAS-cog score, NPI score, and glucose or insulin metabolism. Plasma levels of resveratrol and its metabolites at 45 weeks were approximately: resveratrol 22 ng/mL, 3-*O*-glucuronidated resveratrol 3800 ng/mL, 4-*O*-glucuronidated resveratrol 5000 ng/mL, sulphated resveratrol 7400 ng/mL. The levels in CSF were approximately: resveratrol 0.45 ng/mL, 3-*O*-glucuronidated resveratrol 8.5 ng/mL, 4-*O*-glucuronidated resveratrol 12 ng/mL, sulphated resveratrol 13 ng/mL. The altered CSF Aβ_40_ path and the pharmacokinetic data suggest that the drug penetrates the blood–brain barrier to have central effects; however, the authors concluded that this result must be interpreted with caution, because although it is suggestive of CNS effects, it does not indicate benefit.

Recently, Wong et al. [116] carried out a randomized, double blind, placebo controlled, crossover clinical trial (registered as ACTRN12614000891628) to evaluate the effect of resveratrol supplementation on cerebrovascular function, in adults with a diagnosis of type 2 diabetes mellitus (T2DM). To manage T2DM, volunteers were using diet or exercise alone, or oral hypoglycemic agents. Enrolled participants (38 subjects of both sexes 40–80 years old) were randomly allocated to receive placebo or the resveratrol supplement Resvida™, at doses of 75 mg, 150 mg, or 300 mg, taken as a single dose during four intervention visits that took place at seven day intervals over 4 weeks. The main outcome was to determine the most effective dose of resveratrol to improve the cerebral vasodilator responsiveness (CVR) to hypercapnia in the middle cerebral artery, using Transcranial Doppler ultrasound. Effect of resveratrol on CVR in the posterior cerebral artery was the secondary outcome. Cerebral blood flow velocities were also measured. Any symptom of illness appearing during the trial was recorded. Thirty six participants concluded the study, while two withdrew their consent to participate before the first intervention visit. Resveratrol consumption significantly increased CVR in the middle cerebral artery at all tested doses with maximum improvement being observed with the lowest dose. CVR in the posterior cerebral artery was increased only at 75 mg. No adverse events occurred during the intervention.

Interestingly, several clinical trials evaluating the efficacy of resveratrol, alone or in combination with other supplements, in AD or MCI, are currently at various stages of completion. A randomized, double-blind, placebo-controlled phase three study (NCT00743743) [117] was being designed in order to determine the effects on cognitive and global functioning in patients with mild-to-moderate AD on standard therapy. Fifty patients (50–90 years old) had to be enrolled in the study. The treatment group had to receive 1 capsule daily for 52 weeks of Longevinex brand resveratrol supplement, containing 215 mg of resveratrol active ingredient, while the control group 1 capsule of placebo. However, this study was withdrawn prior to enrolment.

Another randomized, double-blind, placebo-controlled phase 3 study (NCT00678431) [118] recruited 27 subjects of both sexes (50–90 years old) with mild-to-moderate AD. The treatment group received liquid resveratrol (dose not reported) together with glucose and malate, using grape juice as the delivery system. The aim of this trial was to investigate the ability of resveratrol to slow the progression of AD. This endpoint was measured by the ADAS-Cog scale and Clinical Global Impression of Change (CGIC) scale at regular intervals up to 1 year after the study’s beginning. Also, the adverse effects reported by patients were examined. Although this trial was completed in June 2011, results are not available at the moment.

Additionally, a randomized, double-blind, placebo-controlled phase 1 study (NCT01126229) [119] was aimed to assess the longer-term safety (3 months) and efficacy of resveratrol supplementation on cognitive function and physical performance in older adults. Thirty-two participants, of both sexes and aged 65–100 years, were enrolled. Subjects were randomly assigned with equal probability to receive either resveratrol (300 mg/day or 1000 mg/day) or placebo for 12 weeks. A follow-up evaluation was provided at 10 and 30 days following completion of the final post-treatment assessment. At the moment, the results on resveratrol safety have been published [120]; conversely, those about the effects on cognitive function and physical performance are still not available.

In the NCT01794351 [121] clinical trial (randomized, double-blind, placebo-controlled, cross-over study), the potentially cognitive enhancing effects of 500 mg *trans*-resveratrol in 50 healthy young humans (both sexes, 18–35 years old) were evaluated. In this study, participants received firstly placebo and then resveratrol, on separate days, with a 7–14 day wash-out period between visits and, in the second part of the study, first resveratrol and then placebo. The main outcome was to measure the number of participants with altered cognitive function which differed from the baseline, in a time range between 40 min and 6 h post-dose. The secondary outcome was to measure the number of participants with altered mood which differed from the baseline (time range 40 min–6 h post-dose). This study was completed in December 2012, but still results are not available.

Another larger double-blind, placebo-controlled phase four clinical trial (NCT01219244) [122] investigating whether dietary modification could provide positive effects on brain functions in elderly people with MCI is still recruiting participants. The researchers of this study planned to enrol 330 subjects (50–80 years old) with MCI. Participants will be randomly divided in 4 groups receiving placebo, or omega-3 supplementation, or resveratrol supplementation or they will undergo caloric restriction, for 6 months. The study plans also a second step in which the most effective dietary intervention will be combined with physical and cognitive training in order to highlighted the enhancing of memory functions. The effect of dietary modification on brain functions will be measured by ADAs-Cog scale. Moreover, functional and structural brain changes and plasma biomarkers will be evaluated prior to intervention and after 6 months of intervention.

The randomized double-blind placebo-controlled clinical trial NCT01766180 [123] has been designed to determine whether the intake of resveratrol (Resvida; resveratrol 150 mg/day), alone or in combination with Fruitflow-II (tomato extract; 150 mg/day), for a period of 3 months, is effective in improving memory. A cohort of 80 subjects (both sexes aged 50–80 years) with memory impairment will be enrolled. The effects of the medications on brain blood flow and fitness will be also evaluated to find out whether the possible improvement in memory is associated with the alterations in these parameters. Memory improvement will be measured by CANTAB (Cambridge Neuropsychological Test Automated Battery), maximal oxygen uptake and blood flow to the brain. At the moment, the present clinical trial is recruiting participants.

The clinical trial NCT02621554 [124] (randomized, double-blind, placebo-controlled phase 2/3 study) will investigate whether resveratrol could provide positive effects on memory and brain structures and functions in healthy elderly participants (both sexes ≥50 years old) with subjective memory complaints. Resveratrol (exact dose not reported) will be administered to the subjects for 6 months. Primary and secondary outcomes will be used to evaluate changes from baseline, after 6 and 12 months, with Verbal Learning Task Scores, MMSE score, structural and functional changes on the brain MRI images and plasma biomarkers. The present clinical trial is recruiting participants.

Additionally, a randomized double-blind clinical trial phase 1 (NCT02502253) [125] will evaluate the effects of a Bioactive Dietary Polyphenol Preparation (BDPP), a combination of three nutraceutical preparations (grape seed polyphenolic extract, resveratrol, and Concord grape juice) in patients with MCI and prediabetes. Forty-eight subjects of both sexes and aged 55–85 years will be enrolled. Participants will receive for 4 months BDPP preparation at low, moderate and high doses (the exact concentrations of BDPP are not reported in the website of clinicaltrial.gov). The main outcomes of this study will be to assess adverse events and serious adverse events, to confirm brain penetration of BDPP by measuring levels of its constituents in CBF, to evaluate the BDPP effect on mood with NPI and Cornell Scale for Depression in Dementia, and to assess the BDPP effect on cognition with a battery of memory, executive function, and attention measures. The present clinical trial is recruiting participants.

Finally, the study ACTRN12611001288910 [126] (placebo-controlled, double-blind, randomized, phase 1/2, crossover study) is aimed at evaluating the effect of resveratrol in red wine on cognitive function in older adults. Participants (20 healthy subjects ≥65 years old) will be randomized to receive either 100 mg of grape derived resveratrol in 100 mL of red wine and 100 mL of red wine, such that at the conclusion of the study all participants will have received both treatments. The main outcomes will be to assess the effects of a moderate daily amount of resveratrol-enriched red wine on cognitive performance in older adults using the Cognitive Demand Battery (CDB) and to establish whether the dose of resveratrol (100 mg) is significant enough to reach detectable concentrations in the body. At present, the recruitment has been completed.

## 5. Discussion

Our search retrieved five published clinical trials on curcumin and six on resveratrol, investigating their efficacy in preserving or restoring cognitive function. In the range of doses used and relatively to the duration of studies, curcumin and resveratrol appear to be generally safe and well tolerated.

Among the curcumin trials, three were performed in adult or old patients with AD of various degrees; among these, only one [98] was found to have positive effects on AD symptoms: it is worthwhile to point out that this study reported only three single cases. Two studies [99,100] enrolled healthy subjects and only in one of them [99] an improvement in cognitive function was observed; conversely, no effect was observed by Rainey-Smith et al. [100] in their one year long study.

Among the resveratrol studies, only one [115] enrolled patients with AD (mild-to-moderate); in this, a decrease of Aβ_40_ and CSF plasma levels was observed but with no improvement in cognitive score. The other five studies enrolled young, healthy subjects or adult/old subjects with obesity or diabetes mellitus type 2. Results showed an increase in cerebral blood flow but cognitive function was not generally affected, except in one case [113]. Then, present data do not allow to draw conclusions on the efficacy of curcumin and resveratrol in neurodegenerative disorders.

Curcumin and resveratrol possess multitargeting biological effects, both in vitro and in vivo, such as inhibition of Aβ aggregation, reduction of oxidative stress, promotion of cell growth, inhibition of cholinesterase activity, inhibition of brain pro-inflammatory responses, prevention of neuronal cell death, enhancement of neuroprotective sirtuin-1 activity, etc., [8,130] that make them ideal candidates in the battle against neurodegenerative diseases. However, despite their attractive neuroprotective properties, the results obtained in clinical trials are generally disappointing: what is the reason for the discrepancy between pre-clinical and clinical data? Different points deserve to be taken into account in this regard.

First of all, the number of available studies is small and their experimental design is different. The number of subjects enrolled is generally small, too (<50 in eight out of eleven studies published); the duration of follow-up is <6 months in six out of 11 trials and sometimes less than one month, which is inadequate to detect potential changes in cognitive function. A further variable is represented by the dose, particularly as regards curcumin. Doses of curcumin used in the trials vary greatly: from 80 mg/day to 4 g/day. Of note, in patients with AD, Hishikawa et al. [98] observed an improvement of behavioral symptoms and quality of life with curcumin 100 mg/day. Analogously, in healthy subjects, Cox et al. [99] observed an improvement of cognitive functions with curcumin 80 mg/day (as Longvida^®^ Optimized Curcumin). Instead, Baum et al. [96] administrating curcumin at 1 g/day and 4 g/day did not highlight any positive effect. These authors did not even find difference in curcumin plasma levels between 1 g/day and 4 g/day, suggesting that the use of curcumin at doses higher than one gram is not justified. Resveratrol doses used in the trials are generally in the range of between 250 mg/day and 500 mg/day.

Besides the dose, the clinical effect of a substance depends on its bioavailability, then on its formulation: this is a crucial point when talking about curcumin and resveratrol. In clinical trials, these substances are generally administered by oral route. Both curcumin and resveratrol have low water solubility and after oral administration, their absorption is very low (<1%), as reported in the clinical trial above described and in literature [131,132]. After absorption, the substances are metabolized mainly in glucuronides and sulfates while the plasma levels of parent compounds are very low [22]. Poor absorption, rapid metabolism within the human gastro-intestinal tract and rapid systemic elimination result in poor systemic bioavailability of curcumin and resveratrol: this has been the primary challenge to their clinical application [133,134,135]. To overcome these problems, different delivery systems, like adjuvants, nanoparticles, liposomes, micelles, phospholipid complexes and nanogels, have been investigated or are being developed as strategies to improve the bioavailability of the two polyphenols. Among the adjuvants, piperine, a known inhibitor of hepatic and intestinal glucuronidation, has been shown to strongly increase the bioavailability of curcumin and resveratrol when co-administered [136,137]. The solid lipid curcumin particle preparation, commercially available as Longvida^®^, has been reported to give increased bioavailability compared with a generic curcumin [138]. Theracurmin^®^ is a nanoparticle-based curcumin preparation with a greater than 30-fold increase in bioavailability, compared with conventional curcumin [139]. The patented formulation Biocurcumax™ is reported to give a curcumin bioavailability of about 6.93-fold greater than normal curcumin owing to the synergism between the sesquiterpenoids present in turmeric and the curcuminoids [140]. In the formulation Longevinex^®^, resveratrol is supplemented with 5% quercetin and 5% rice bran phytate: these ingredients are micronized to increase the bioavailability. Resveratrol is sometimes administered in red wine to increase its bioavailability. In fact, the pharmacokinetics of resveratrol may dramatically change depending on the food matrix in which it is found: when it is part of wine uptake it is higher than the pure compound [134].

Noteworthy, some of the preparations mentioned above have been used in the clinical trials that gave positive results. For instance, Cox et al. [99] used the formulation Longvida^®^ while Hishikawa et al. [98] administered turmeric as a source of curcumin. Studies have indicated that turmeric oil, present in turmeric, can enhance the bioavailability of curcumin. Moreover, some individual components of turmeric, including turmerin, turmerone, elemene, furanodiene, curdione, bisacurone, cyclocurcumin, calebin A, and germacrone possess biological activity that can potentiate the beneficial effects of curcumin [141]. In addition, Witte et al. [113] obtained a significant retention of memory by administering resveratrol in a formula with quercetin, while Wightman et al. [114] observed that piperine enhances the bioavailability of resveratrol and its effect on cerebral flow, even if the cognitive performance was not affected. We can then suppose that the use of novel formulations can allow to better highlight the possible clinical efficacy of curcumin and resveratrol.

Besides the problems above discussed, it has to be considered that Aβ-induced alterations occur in the earliest stages of AD. When symptoms occur there has already been a substantial loss of neurons, so it is only possible to counteract the symptoms; this is the reason for the limited efficacy of current pharmaceutical drugs and, probably, of the failure of curcumin and resveratrol. Then, it emerges in a compelling way the need for an early diagnosis of the disease that, in turn, requires the identification of suitable peripheral biomarkers, not only in the blood but also in other districts. In this context, the ongoing studies using curcumin in the early diagnosis of AD are of particular interest. This approach is based on the ability of curcumin to fluorescence and to bind Aβ both in the brain and in the retina [142,143]. Since the pathology in the retina can be detected before it could be detected in the brain, curcumin can represent a useful pre-clinical biomarker.

Furthermore, future studies aimed at highlighting the ability of new substances to prevent AD or to slow the progression of cognitive decline should be carried out in healthy older subjects and should have a long-lasting follow-up.

Finally, new drugs able to act in the early stages of AD are needed. Curcumin and resveratrol are pleiotropic substances that interfere with numerous pathophysiological mechanisms that precede the onset of AD, then they could be more likely effective at the earliest pre-clinical stages, before onset of symptoms and could act by slowing the disease progression.

As already stated, several clinical trials on curcumin and resveratrol are ongoing, most of them using curcumin and resveratrol formulations with high bioavailability; it is hoped that these trials provide more information on the possible efficacy of curcumin and resveratrol in preserving or restoring the cognitive function. Also, these, however, present some critical points, particularly as regards the standardization of products. For example, in the trial ACTRN12613000367741 [108], the treatment reported is “curcumin 20 g/day” but probably the dose is referred to the whole supplement (Longvida^®^) which contains 20% of curcumin, corresponding to 4 g of active substance. In the trial NCT02502253 [125], the treatment Bioactive Dietary Polyphenol Preparation (BDPP), a combination of grape seed polyphenolic extract [GSE], resveratrol, and Concord grape juice, is administered at low, moderate and high doses, but neither the doses nor the amount of resveratrol contained in the preparation is described. In order to obtain repeatable data it should be mandatory to use well standardized products: this should always be kept in mind in the search for natural products, particularly when they are used in the form of phytocomplexes.

## 6. Conclusions

AD is a multifactorial disorder that requires drugs capable of operating on multiple brain targets. Polyphenols, particularly curcumin and resveratrol, having pleiotropic protective effects appear to be ideal candidates to prevent or treat neurodegenerative disorders, however, their clinical efficacy and utility is still an open question. In order to exploit the protective properties of these compounds, some key points should be addressed.

Considering the scarce efficacy of current pharmacological treatments, the goal should be an early diagnosis of the pathology and intervention should be aimed at preventing or slowing down its progress. In this context, it appears of primary importance to identify suitable biomarkers, particularly blood biomarkers, easy to obtain and that can be repeated as necessary.

Curcumin and resveratrol, owing to their capability to interfere with a series of processes implicated in the early stages of pathology, when clinical symptoms have not yet appeared, could be useful in preventing neurodegeneration or slowing it down. However, it is of primary importance to establish the effective dose, improve the bioavailability of substances and use well standardized preparations, capable of ensuring plasma levels sufficient for the protective action. Considering that curcumin and resveratrol are generally marketed as food supplements and that for each substance exists a wide variety of preparations, the bioavailability of the different formulations should be assayed comparatively.

Finally, the potential preventive activity of curcumin and resveratrol should be evaluated in long-term exposure clinical trials, using specific designs, different from therapeutic effect trials. Such information is also requested by authorities like the FDA (Food and Drug Administration) and EFSA (European Food Safety Authority) to support health claims.

## Figures and Tables

**Table 1:** Clinical trials on the effects of curcumin on cognitive function.

Reference and/or ID (Location)	Study Design Phase	Curcumin Preparation and Dose [Other Medication]	Duration	Subjects *n* Age Disorder/Status	Primary Purpose	Main Results	Adverse Events	Status
Baum et al. [96] (Hong Kong, China) NCT00164749	R, DB, PC NR	Curcumin 1 or 4 g/day (standardized ginkgo extract 120 mg/day)	6 months	34 ≥50 years Probable or possible AD	Effect in AD	No significant differences between curcumin and PL	No differences between curcumin and PL	Published
Ringman et al. [97] (Westwood, CA, USA) NCT00099710	R, DB, PC Phase 2	Curcumin C3 Complex^®^ 2 or 4 g/day (1.9 or 3.8 g/day curcuminoids) ^a^ (AchE-Is and memantine)	24 weeks with an open-label extension to 48 weeks	36 ≥49 years Mild-to-moderate AD	Safety and efficacy with regard to cognition	No significant differences between curcumin and PL	No significant differences between curcumin and PL	Published
Hishikawa et al. [98] (Kariya, Japan)	Single cases	Turmeric 764 mg/day (curcumin 100 mg/day) (Yokukansan 2/3 subjects; donezepil 3/3)	12 weeks	3 79–83-84 years Progressive dementia	Effect on some symptoms of AD	Improvement in behavioral symptoms and quality of life	NR	Published
Cox et al. [99] (Melbourne, Australia) ACTRN12612001027808	R, DB, PC Phase 3/Phase 4	Longvida^®^ Optimized Curcumin 400 mg (80 mg curcumin)	4 weeks	60 60–85 years Healthy	Effect on cognitive function	Improvement of cognitive functions	Treatment was well tolerated	Published
Rainey-Smith et al. [100] (Jondalup, Australia) ACTRN12611000437965	R, DB, PC	BCM-95^®^CG (Biocurcumax™) 1500 mg/day (1320 mg/day curcuminoids)	12 months	160 40–90 years Healthy	Prevention of cognitive decline	No changes in cognitive performance	Gastrointestinal complaints in 23 subjects	Published
NCT00595582 [101] Shreveport, LA, USA	Open-label NR	Curcumin 5.4 g/day (bioperine)	24 months	10 55–85 years MCI or mild AD	Effect on MCI or mild AD	None patient terminated the study	Two patients showed dyspepsia	Terminated
NCT01001637 [102] (Mumbai, Maharashtra, India)	R, DB, PC Phase 2	Longvida^®^ 4 or 6 g/day	2 months	26 50–80 years Probable AD	Safety and effect on AD	-------	-------	Unknown
NCT01383161 [103] (Los Angeles, CA, USA)	R, DB, PC Phase 2	Theracurmin™ 2.79 g/day (180 mg/day curcumin)	18 months	132 50–90 years MCI	Effect on age-related cognitive impairment	-------	-------	Active, not recruiting
NCT01811381 [104] (Los Angeles, CA, USA)	R, DB, PC Phase 2	Longvida Curcumin^®^ (800 mg/day of curcumin)	12 months	80 55–90 years MCI	Effect of curcumin alone or combined with yoga on AD	-------	-------	Recruiting
ACTRN12616000484448 [105] (Hawthorn, Australia)	R, DB, PC Phase 3/Phase 4	Longvida^®^ Optimized Curcumin 400 mg/day (80–90 mg curcumin)	12 weeks	80 50–85 years Healthy	Effects on cognition, mood and well-being	-------	-------	Not yet recruiting
ACTRN12613000681752 [106] (NSW, Australia)	R, DB, PC Phase 2	Curcumin (Biocurcumax™): from 500 mg/day then 1 g/day then 1.5 g/day onwards	12 months	100 65–90 years Healthy but at high risk of AD	Prevention of AD	-------	-------	Recruiting
ACTRN12614001024639 [107] (sub study of ACTRN12613000681752) [106] (NSW, Australia)	R, B, PC Phase 2	Curcumin 1.5 g/day	3–6 months	48 65–90 years Healthy and MCI	Influence on the expression of inflammatory genetic markers	-------	-------	Not yet recruiting
ACTRN12613000367741 [108] (Nedlands, Australia)	Open study, not randomized Phase 2	Curcumin 20 gm/day (Vitamin E 500 IU/day)	7 days	40 ≥50 years AD, MCI, and healthy	Earlier detection of AD	-------	-------	Not yet recruiting
ACTRN12615000465550 [109] (Nedlands, Australia)	Open study, not randomised, unblended Phase 2/Phase 3	Longvida^®^ 20 g/day (4 g/day curcumin) (Vitamin E 500 IU/day for 8 days)	7 days	100 ≥50 years Healthy and MCI	Earlier detection of AD	-------	-------	Recruiting
ACTRN12615000677505 [110] (Nedlands, Australia)	Open study, not randomised, Phase 2	Longvida^®^ 20 g/day (containing 4 g curcumin)for 7 days (Vitamin E 500 IU/day for 8 days)	7 days	40 40–60 years Healthy	Earlier detection of AD	-------	-------	Not yet recruiting

**^a^** 95% curcuminoids with curcumin 70%–80%, demethoxycurcumin 15%–25%, bisdemethoxycurcumin 2.5%–6.5%. ID, Identifier; NR, Not Reported; R, randomized; DB, double blind; B, blind; PC, placebo controlled; AD, Alzheimer disease; PL, placebo; AchE-Is, Acethylcholinesterase inhibitors; MCI, Mild Cognitive Impairment; AEs, adverse events; SAEs, serious adverse events.

**Table 2:** Clinical trials on the effects of resveratrol on cognitive function.

Reference or ID (Location)	Study Design	Resveratrol Preparation and Dose [Other Medication]	Duration	Subjects *n* Age Disorder/Status	Purpose Outcome Measures	Main Results	Adverse Events	Status
Kennedy et al. [111] (Newcastle upon Tyne, UK)	R, DB, PC, CO	*Trans*-resveratrol from Biotivia Bioceuticals (Vienna, Austria) 250 mg or 500 mg	21 days	24 18–25 years Healthy 9 further subjects underwent bioavailability assessment	To investigate the ability to modulate mental function and increase cerebral blood flow	Cognitive function not affected. Increase in cerebral flow	Not assessed	Published
Wong et al. [112] (Adelaide, Australia) ACTRN12611000060943	R, DB, PC, CO	Resvida (resveratrol 75 mg/day)	12 weeks	28 45–70 years Obese but otherwise healthy	Effects of resveratrol on circulatory function and cognitive performance in obese adults	Increase of circulatory function. No effects on blood pressure, arterial compliance, and cognitive function	Resveratrol appears safe and well tolerated	Published
Witte et al. [113] (Berlin, Germany)	R, DB, PC,	Resveratrol 200 mg/day in a formula with quercetin	26 weeks	46 50–80 years Healthy overweight	To investigate the ability to enhance cognitive performance	Significant retention of memory, significant increase of hyppocampal FC, improvement of glucose metabolism	Not assessed	Published
Wightman et al. [114] (Newcastle upon Tyne, UK)	R, DB, PC, CO	*Trans*-resveratrol 250 mg/day or *trans*-resveratrol 250 mg/day with 20 mg piperine	21 days	23 19–34 years Healthy 6 healthy men underwent bioavailability assessment	To assess if piperine affects the efficacy and bioavailability of resveratrol	Piperine henances the effect of resveratrol on cerebral blood flow but not the cognitive performance and bioavailability	Not assessed	Published
Turner et al. [115] (Georgetown, USA) NCT01504854	R, DB, PC, MC Phase 2	Resveratrol 500 mg/day with dose excalation by 500 mg increments ending with 2 g/day	52 weeks	119 >49 years Mild-to-moderate AD	To assess safety and efficacy	Decrease of CSF and plasma Aβ_40_ levels. No significant effects on cognitive score	Resveratrol appears safe and well tolerated	Published
Wong et al. [116] ACTRN12614000891628 (Newcastle, Australia)	R, DB, PC, CO Phase 2	Resvida 75 mg/day, 150 mg/day, 300 mg/day	4 weeks	36 40–80 years Type 2 diabetes mellitus	Improvement of cerebrovascular responsiveness	Increase of cerebrovascular responsiveness	None	Published
NCT00743743 [117] (Milwaukee, WI, USA)	R, DB, PC Phase 3	Longevinex brand resveratrol supplement (resveratrol 215 mg/day)	52 weeks	50 50–90 years Mild-to-moderate AD on standard therapy	Effects on cognitive and global functioning	-------	-------	Withdrawn prior to enrollment
NCT00678431 [118] (Bronx, NY, USA)	R, DB, PC Phase 3	Resveratrol with glucose and malate	12 months	27 50–90 years Mild-to-moderate AD	To assess the ability to slow the progression of AD	Not available	Not available	Completed in June 2011
NCT01126229 [119] (Gainesville, FL, USA)	R, DB, PC Phase 1	Resveratrol 300 mg/day or 1000 mg/day	12 weeks	32 ≥65 years old	To assess the longer-term safety (3 months) and efficacy on age-related health conditions	Not available	Resveratrol appears safe and well tolerated [120]	Completed in December 2012
NCT01794351 [121] (Newcastle upon Tyne, UK)	R, DB, PC, CO	*Trans*-resveratrol 500 mg in unique dose	14 days	50 18–35 years Healthy	To assess the potential cognitive enhancement	Not available	-------	Completed in December 2012
NCT01219244 [122] (Berlin, Germany)	R, DB, PC Phase 4	Resveratrol or omega-3 supplementation or caloric restriction	6 months	330 50–80 years MCI	Effects on brain functions	-------	-------	Recruiting
NCT01766180 [123] (Lutherville, MD, USA)	R, DB, PC	ResVida (resveratrol 150 mg/day) alone or associated with Fruitflow ^a^-II 150 mg/day	3 months	80 50–80 years Subjects with memory impairment	Efficacy in treating memory problems	-------	-------	Recruiting
NCT02621554 [124] (Leipzig, Germany)	R, DB, PC Phase 2/Phase 3	Resveratrol (dose not reported)	12 months	60 ≥50 years Healthy or with subjective memory complaints	Effects on memory and on brain structures and functions	-------	-------	Recruiting
NCT02502253 [125] (Baltimore, MD, USA)	R, DB Phase 1	Bioactive Dietary Polyphenol Preparation (BDPP) at low, moderate and high dose	4 months	48 55–85 years MCI	Safety and efficacy in treating mild cognitive impairment and prediabetes	-------	-------	Recruiting
ACTRN12611001288910 [126] (Hawthorn, Australia)	R, DB, PC, CO Phase 1/Phase 2	100mg of grape derived resveratrol in 100ml red wine	8 days (washout 7 days)	20 ≥65years Healthy	To assess the effect of resveratrol in red wine on cognitive function in older adults	-------	-------	Recruitment completed

^a^ tomato extract. ID, Identifier; AD, Alzheimer disease; MCI, Mild Cognitive Impairment; R, randomized; DB, double blind; PC, placebo controlled; CO, cross over; MC, multicenter; FC, functional connectivity; CSF, Cerebrospinal fluid.

## Abbreviations

The following abbreviations are used in this manuscript:

AD: Alzheimer disease
ADAS-Cog: Alzheimer’s Disease Assessment Scale-Cognitive subscale
ADCS-ADL: Alzheimer’s Disease Co-operative Study-Activities of Daily Living
AEs: Adverse Events
Aβ: Amyloid β
AUC: Area under the curve
BDPP: Bioactive Dietary Polyphenol Preparation
CANTAB: Cambridge Neuropsychological Test Automated Battery
CBF: Cerebral Blood Flow
CDB: Cognitive Demand Battery
CDR: Clinical Dementia Rating
CGIC: Clinical Global Impression of Change
CVR: Cerebral Vasodilator Responsiveness
DNMT1: DNA Methyltransferase
EFSA: European Food Safety Authority
FDA: Food and Drug Administration
FDG-PET: 2-Deoxy-2-[fluorine-18]fluoro-d-glucose Positron Emission Tomography
FMD: Flow Mediated Dilatation
fMRI: functional Magnetic Resonance Imaging
HAT: Histone Acetyltransferases
HO-1: Heme oxygenase-1
iNOS: Inducible Nitric Oxide Synthase
MAPK: p38 Mitogen-activated protein kinase
MCI: Mild Cognitive Impairment
MMSE: Mini-Mental Status Examination
NF-kB: Nuclear factor kappa-light-chain-enhancer of activated B cells
NIRS: Near-Infrared Spectroscopy
NPI: Neuropsychiatric Inventory
NPI-Q: Neuropsychiatric Inventory-brief Questionnaire
PET: Positron Emission Tomography
RAI: Retinal Amyloid Index
RNS: Reactive Nitrogen Species
ROS: Reactive Oxygen Species
SAEs: Serious Adverse Events
SIRT-1: Sirtuin-1
STAT1: Signal Transducer and Activator of Transcription 1
STAT3: Signal Transducer and Activator of Transcription 3
TNF-α: Tumour Necrosis Factor α
